# A Growth Curve Model with Fractional Polynomials for Analysing Incomplete Time-Course Data in Microarray Gene Expression Studies

**DOI:** 10.1155/2011/261514

**Published:** 2011-09-27

**Authors:** Qihua Tan, Mads Thomassen, Jacob v. B. Hjelmborg, Anders Clemmensen, Klaus Ejner Andersen, Thomas K. Petersen, Matthew McGue, Kaare Christensen, Torben A. Kruse

**Affiliations:** ^1^Department of Clinical Genetics, Odense University Hospital, Sdr. Boulevard 29, 5000 Odense C, Denmark; ^2^Epidemiology and Department of Biostatistics, Institute of Public Health, University of Southern Denmark, J. B. Winsløws Vej 9B, 5000 Odense C, Denmark; ^3^Department of Dermatology and Allergy Center, Odense University Hospital, Sdr. Boulevard 29, 5000 Odense C, Denmark; ^4^Discovery, LEO Pharma A/S, Industriparken 55, 2750 Ballerup, Denmark; ^5^Department of Psychology, University of Minnesota, Minneapolis, MN 55455, USA

## Abstract

Identifying the various gene expression response patterns is a challenging issue in expression microarray time-course experiments. Due to heterogeneity in the regulatory reaction among thousands of genes tested, it is impossible to manually characterize a parametric form for each of the time-course pattern in a gene by gene manner. We introduce a growth curve model with fractional polynomials to automatically capture the various time-dependent expression patterns and meanwhile efficiently handle missing values due to incomplete observations. For each gene, our procedure compares the performances among fractional polynomial models with power terms from a set of fixed values that offer a wide range of curve shapes and suggests a best fitting model. After a limited simulation study, the model has been applied to our human *in vivo* irritated epidermis data with missing observations to investigate time-dependent transcriptional responses to a chemical irritant. Our method was able to identify the various nonlinear time-course expression trajectories. The integration of growth curves with fractional polynomials provides a flexible way to model different time-course patterns together with model selection and significant gene identification strategies that can be applied in microarray-based time-course gene expression experiments with missing observations.

## 1. Introduction

The time course experiment is an important experimental design that permeates throughout biomedical research. With the recent popularity of high throughput microarray-based gene expression analysis, the time-course design has been applied to explore global transcriptional responses to treatment or to biochemical stimulations during *in vivo* or *in vitro* experiments. Analysing the time-course microarray gene expression data is a new challenge in bioinformatics and biostatistics [1–6]. Different from ordinary time-course studies that focus on one or a limited number of outcome variables, the array-based time-course experiment measures expression levels for thousands of genes simultaneously [7]. This complicates the model fitting process because it is impossible to inspect the observed and the fitted time-course patterns for determining a proper parametric form for the model (e.g., the order of a power polynomial), for each of the thousands of genes measured on the arrays. Model selection can be tedious given the various response patterns for different genes which cannot be predefined. Besides the above characteristics in an array-based experiment, time-course gene expression data are also featured by occasional missing measurements during the experiment resulting in incomplete observations due to various reasons (experimental, technical, material, etc.). Making use of incomplete data is an important issue in time-course data analysis [8] because simply discharging incomplete observations can result in reduced power and even biased estimates of parameters. 

The growth curve model is a popular modern model for analyzing sequential follow-up data collected in epidemiological studies using a longitudinal design [9]. Such experiments are featured by the repeated measurements on the same subject over the follow-up time. In these experiments, the researchers are interested in making inferences on the growth and change patterns in various time-related measurement variables, for example, physical and cognitive functions during aging [10], changes in BMI [11], status of health over time [12], and recovery from diseases [13]. The growth curve analysis models each individual's profile by estimating individual specific slope and intercept parameters allowing for the study of different aspects of the process of change concerning patterns of change (linear or nonlinear) together with their variance and covariance [14]. The individualized parameter estimation in the growth curve modelling process also enables efficient use of incomplete observations which can occur during a time-course experiment. Moreover, the growth curve model allows inclusion of both discrete and continuous covariates in the model to be analysed simultaneously. 

Fractional polynomials (FPs) are an extension of the well-established polynomial method of modelling with continuous variables including time. FPs represent a class of time transformations with power restricted to a special set of positive or negative integers and fractions [15] with attractive features including parsimony, a wide range of curve shapes for low-order models, and the ability to approximate asymptotes. FPs have been integrated in regression models to model nonlinear relationships, for example, in logistic regression [16], in survival analysis [17], and most recently in mixed effect model [18]. The fixed set of exponents in FPs enables automatic model selection for the best fitting model through model performance comparison for each of the genes in a microarray study. 

Generally, researchers conducting a longitudinal epidemiological study or a time-course microarray experiment share a common interest, that is, exploring the various features of change over time. Although the duration of time for an array-based laboratory experiment is usually much shorter (can be in hours) than that in a longitudinal survey (can be in years); however, this should analytically make no difference in terms of growth curve modelling. With this consideration, we exemplify application of the growth curve model with FPs in the analysis of microarray time-course expression data with missing observations from an experiment of human *in vivo* irritated epidermis [19]. We show that our procedure can automatically identify the significant time trajectories in gene regulatory response through model comparison and statistical testing while efficiently handling missing values.

## 2. Methods

### 2.1. The Growth Curve Model with Fractional Polynomials

Starting from time 0, a microarray time-course experiment measures the expression level for a large number of genes for individual *i*  (*i* = 1,2, 3,…, *N*) at time point *j*  (*j* = 1,2, 3,…, *n*_*i*_) which we designate as an *n*_*i*_ × 1 individual time series vector **Y**_**i**_ for one of the genes on the array. The expression level over time can be expressed at individual level as

(1) $$Y_{i}=X_{i}\beta +Z_{i}b_{i}+e_{i}.$$

Here **X**_**i**_ is the design matrix of size *n*_*i*_ × (*p* + 1) with (*p* + 1) being the number of terms of fixed effect including the intercept; **β** is a (*p* + 1) × 1 vector of regression coefficients that are to be estimated by the model; **Z**_**i**_ is a matrix of size *n*_*i*_ × (*q* + 1) with (*q* + 1) the number of terms of random effect, that links **b**_**i**_ the unobserved random effects with **Y**_**i**_, and **e**_**i**_ is an *n*_*i*_ × 1 unobserved vector of random errors for the *i*th individual. Here, **b**_**i**_ ~ *N*(0, **G**), where **G** is a (*q* + 1)(*p* + 1) unknown variance-covariance matrix of random effects to be estimated; **e**_**i**_ ~ *N*(0, **R**_**i**_) with **R**_**i**_ = *σ*_*e*_^2^ **I**_*n*_*i*__  and **I**_*n*_*i*__  an *n*_*i*_ × *n*_*i*_ identity matrix. 

To simplify the description, we assume that each fixed effect has a corresponding random effect, that is, *p* are *q* equal (in practice *q* ≤ *p*) such that

(2) $$X_{i}=Z_{i}=\left[\begin{array}{ccc} {1\;\;\;\;f}_{1}\left(t_{i,1}\right) & \cdots & f_{p}\left(t_{i,1}\right)\\ \vdots & \ddots & \vdots \\ {1\;\;\;\;f}_{1}\left(t_{i,n_{i}}\right) & \cdots & f_{p}\left(t_{i{,n}_{i}}\right) \end{array}\right].$$

In (2), each element is an FP transformation of time with which (1) can be rewritten as

(3) $$y_{ij}=\beta_{0}+\overset{p}{\underset{b=1}{\sum }}\beta_{b}f_{b}\left(t_{i,j}\right)+b_{i0}+\overset{p}{\underset{g=1}{\sum }}b_{ig}f_{g}\left(t_{i,j}\right)+e_{i,j}.$$

Equation (3) expresses the expression level for individual *i* at time point *j* as the sum of fixed effects and random effects from the *p* time transformations in the design matrix, plus an error term. The group equivalence of (3) can be written as

(4) $$E\left(y_{i,j}\right)=\beta_{0}+\overset{p}{\underset{b=1}{\sum }}\beta_{b}f_{b}\left(t_{i,j}\right).$$

Royston and Altman [15] defined the power function *f*_*b*_(*t*_*i*,*j*_) as

(5) $$f_{b}\left(t_{i,j}\right)=\{\begin{array}{cc} t_{i,j}^{\varphi_{b}} & \text{if}\;\varphi_{b}\neq \varphi_{b-1},\\ f_{b-1}\left(t_{i,j}\right)\ln\;\;\left(t_{i,j}\right) & \text{if}\;\varphi_{b}=\varphi_{b-1}, \end{array}$$

where the power term, *φ*_*b*_, can be restricted to a set of values [15]

(6) φ={−2,−1,−0.5,0,0.5,1,2,3}

with *φ*_*b*_ = 0 denoting a natural log transformation of time, that is, ln  (*t*_*i*,*j*_). Although the order of the power function *p* can be any number, practical applications have shown that the second-order (*p* = 2) FP models already offer a wide range of curvature shapes that capture the applied situations [20] with the second-order models for (4) as

(7) $$E\left(y_{i,j}\right)=\{\begin{array}{cc} \beta_{0}+\beta_{1}t_{i,j}^{\varphi_{1}}+\beta_{2}t_{i,j}^{\varphi_{2}} & \text{if}\;\varphi_{1}<\varphi_{2},\\ \beta_{0}+\beta_{1}t_{i,j}^{\varphi_{1}}+\beta_{2}t_{i,j}^{\varphi_{1}}\ln\;\;\left(t_{i,j}\right) & \text{if}\;\varphi_{1}=\varphi_{2}, \end{array}$$

with (*φ*_1_, *φ*_2_) ∈ *φ* × *φ*. Equation (7) represents 36 nonlinear models. By reversing the signs of the *β*s, the shapes of the curves can be flipped in that modelling extensive coverage of time-course patterns can be achieved within a fixed set of power for transformations.

After the transformation of time as described above, the growth curve model with FPs can be estimated using the restricted maximum likelihood (REML) approach implemented in the free *R* package *lme4*.

### 2.2. Best Model Selection

The fixed set of power transformation enables similar model fitting across thousands of genes on the array. We suggest choosing model complexity with consideration of data characteristics (size and number of time points). For each gene, we can fit different models and the best fitting model selected using the maximized likelihood based indices such as the AIC (Akaike Information Criterion) [21] with the lowest AIC for the best model. Significant time-course genes are selected if both *β*_1_ and *β*_2_ are significant in the best-fitting second-order model. Levels of statistical significance are adjusted for multiple testing by calculating the false discovery rate (FDR) [22].

### 2.3. Clustering of Time-Course Patterns

The above procedures identify genes displaying significant monotonous or nonmonotonous time-course expression patterns. It is thus necessary to group these genes into different patterns for further characterization and for visualization. To do that, we apply the popular hierarchical clustering method [23] performed using **R** package *gplots* and the plot function in **R** to examine the various time-course patterns identified using heatmap and time-course plot.

## 3. Simulation Study

We conducted a limited simulation study to examine the performance of the model in dealing with missing observations. To simplify the simulation, we limited the simulation model to order one and fix *φ*_*b*_ to 1 which resulted in a simple linear model comparable to ANOVA. In the simulation, we assigned 6 time points spanning 10 hours and specify 4 groups of genes with fold changes of 2, 2.5, 3, and 3.5, respectively, with each group containing 10 genes. In addition, on each microarray chip, we also assumed that there are 9960 genes with random effects. A total of 20 individuals were generated, and for each of them expression levels of 10,000 genes were measured across the six time points. Statistical significance for each gene tested was adjusted for multiple testing in the large number of genes on the array. For that purpose, we calculated the popular false discovery rate (FDR) [22] and define significance for FDR < 0.1. Different proportions of missing observation (5%, 10%, 25%, 50%, and 70%) were specified and assigned randomly in the 20 individuals and across time points. With this setting, we assessed the power for detecting genes with different fold changes under varying proportion of missing observations.  Table 1 presents the power for different combinations of fold change and proportion of missing estimated from 100 replicates. As can be seen, for the simple linear model, low proportions of missing only have minor effects on power estimates for genes with twofold changes. For genes with more than 3-fold changes, the influence of missing observations is limited even as high as over half of the observations are missing. Overall, our simulation study indicated that the method makes efficient use of incomplete observations in capturing significant time-course patterns.

## 4. Model Application

The epidermal response to chemical irritants was investigated by Clemmensen et al. [19] using genome-wide expression analysis for 47,000 transcripts or genes in a time-course design applied to human *in vivo* irritated epidermis. We apply our method to a subset of their data as an example. In the subsample, epidermal biopsies were taken from 9 human volunteers before and at 0.5, 4, and 24 hours after exposure to sodium lauryl sulphate (SLS). Although biopsies were collected for all 9 participants at initial time, that is, before exposure, complete data were not available for the subsequent experiments resulting in considerable portion of missing observations (Table 2) for which gene expression data were not measured. 

**Figure 1:**
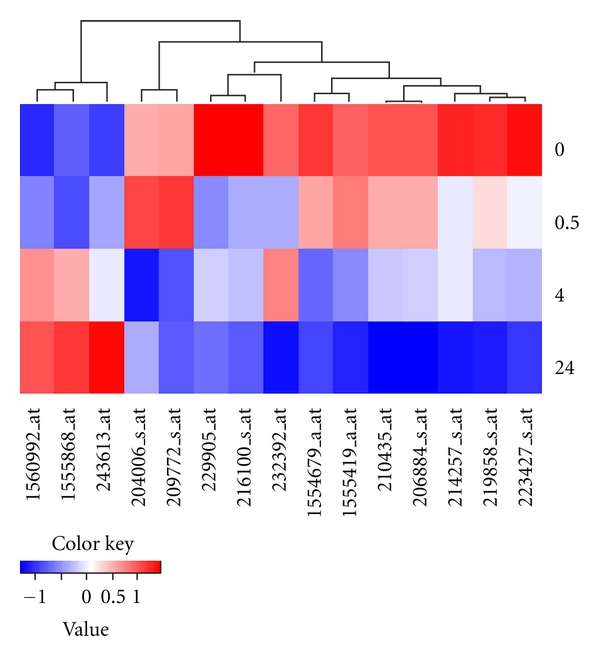
Heatmap showing the mean expression levels for the 15 genes with significant time-course patterns clustered using the hierarchical clustering method and ordered sequentially with time.

**Figure 2:**
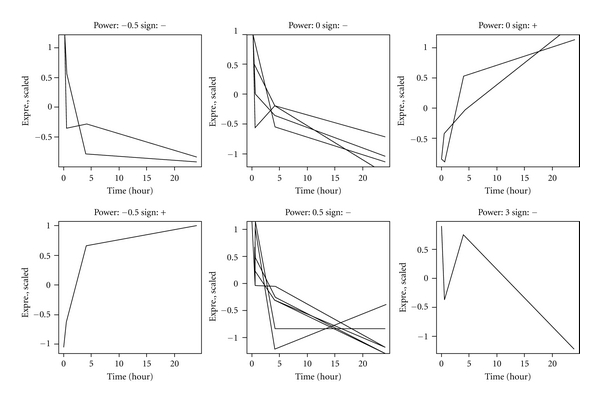
Time-course expression patterns for the 15 significant genes plotted according to the estimated power for transformation and sign of the regression coefficient.

With the data analysis method described above, we fitted the first-order models to each of the genes considering the limited number of time points due to missing observations at individual level. In the analysis, we assumed both random and fixed effects for the intercept and only fixed effect for *β*_1_. Since the values for time start from zero, we added a value of one to each time measurement to facilitate model fitting as power transformation includes log and inverse exponents. AICs were calculated and compared to assign a best fitting model to each gene. For the best fitting model assigned to each gene, we assessed the statistical significances for *β*_1_. A total of 15 genes showed FDR < 0.05.  Figure 1 is a heatmap displaying the identified time-course expression patterns clustered using the hierarchical clustering method. As can be seen in Figure 1, the expression trajectories are dominated by a large cluster of genes to the right of the figure that are downregulated over time (13 genes). These genes are further divided into subclusters depending on their variations in their patterns of decline. In contrast, the small cluster to the left of Figure 1 contains 3 genes that are upregulated during the time-course. In Figure 2, the observed expression patterns for the 15 significant genes are further plotted according to the estimated power of transformation and the sign (− or +) for *β*_1_. As can be seen, the combination of the power and the sign groups the genes into subclusters that correspond to those displayed in Figure 1. Finally we performed a gene ontology enrichment analysis to examine the functional clusters of the identified genes using the online analytical tool g:Profiler at http://biit.cs.ut.ee/gprofiler/. The list of 15 genes give statistically significant enrichment score for a functional cluster of vesicle-mediated transport (*p* = 0.030) and borderline significance for functional groups including protein transport (*p* = 0.055), establishment of protein localization (*p* = 0.056), and membrane (*p* = 0.059).

## 5. Discussion and Conclusions

We have shown through example application, that the growth curve models with fractional polynomials can be applied to analyze time-course microarray gene expression data with incomplete observations. As can be seen, the growth curve analysis of microarray time-course data is characterized by the following features. First, the method provides an elegant way for handling missing observations and makes efficient use of available data by growth curve modelling. Second, the use of fractional polynomials for analysis of microarray data offers a flexible way for capturing various time-dependent expression trajectories for different genes. Third, within a fixed set of exponents for power transformation, the fitting of FPs can be automated and best performance model selected for each gene on the array. This is important because, in practice, it is impossible to manually examine each polynomial pattern fitted across thousands of genes. Finally, for the identified significant time-course genes, our analytical strategy makes use of popular gene clustering methods for time-course pattern characterization and for visualization. As shown by our example application, various time-dependent expression profiles can be revealed and easily perceived.

In theory, nonlinear patterns can be modelled by high-order polynomial functions. High-order modelling can lead to over fitting and at the same time reduces the power of analysis when sample size is limited which is usually the case for most microarray studies. The order two FPs restrict the number of estimating parameters while discriminate the powers for polynomials within a fixed set of power transformations. Although this way of model building could miss the exact polynomial function if it existed, it offers a nice and economic way to capture various nonlinear patterns in small-scale studies. 

The purposes of a microarray time-course experiment are not only identifying significant genes but most importantly how these genes are regulated over time during the experiment. The flexible model selection procedure in our method allows both monotonous and nonmonotonous patterns be fitted through combinations of estimated coefficients for the polynomials and the 36 sets of power transformations which is followed by model selection based on goodness of fit. With help of popular data visualization methods, important time-course patterns for the identified genes can be examined for biological interpretations.

Ernst et al. [24] analyzed the length of time series in microarray time-course studies and found that the published literature is dominated by experiments with small number of time points. We emphasize that the choice of models (first- or second-order models) should be made in consideration of the number of time points in the experiment and proportion of time points missing by patients during the experiment. We suggest using the first-order models (including the simplest linear model) when proportion of missing is high, for example, when only a couple of time points are available at patient level. According to our simulation study, missing observations can strongly reduce the power in detecting genes with relatively low regulation levels. On the other hand, the power for identifying highly regulated genes (over 3-fold changes) is not affected except in extreme situations (more than half observations missing). The fitting of second-order models requires sufficient time points (>3) at patient level to ensure model identification. One should keep in mind that complex time-course patterns can only be captured when sufficient time-points are observed across patients. 

Although not illustrated in our example, it is necessary to mention that, similar to conventional regression analyses, the growth curve model also allows inclusion of covariates in the modelling process. This helps to balance the effects of additional factors that also influence gene expression such that the response trajectories can be more clearly characterized. For example, Tan et al. [25] showed that there are a large number of genes that are differentially regulated by age. Except age, a microarray experiment can be confounded by patients' clinical characteristics, treatment received, and so forth. More importantly, this feature can help to extend growth curve model to microarray studies in, for example, case-control design in time-course experiments, and even account for interaction effects [26]. 

In summary, our proposed method makes use of the nice features of the growth curve model in analyzing time-course expression data especially in dealing with missing observations. The integration of growth curves with fractional polynomials provides a flexible way to model various time-course patterns together with model selection and significant gene identification strategies that can be applied in microarray-based time-course gene expression experiments.

## Figures and Tables

**Table 1:** Power assessment for different proportion of randomly missing observations.

Fold change	Missing proportion					
	0%	5%	10%	25%	50%	70%
2	0.62	0.60	0.53	0.40	0.19	0.08
2.5	0.97	0.97	0.96	0.91	0.64	0.27
3	1.00	1.00	1.00	1.00	0.94	0.58
3.5	1.00	1.00	1.00	1.00	1.00	0.83

**Table 2:** The incomplete structure of example data. Many observations were missing during the time-course experiment for which gene expression data were not available. A total of 21 observations were available from 9 subjects.

	Time (hour)			
	0	0.5	4	24
Subject				
1	×		×	×
2	×	×	×	
3	×	×		
4	×	×		
5	×	×		
6	×	×		×
7	×	×		
8	×		×	×
9	×			
